# Is Alcohol Use Disorder Identification Test (AUDIT) or Its Shorter Versions More Useful to Identify Risky Drinkers in a Chinese Population? A Diagnostic Study

**DOI:** 10.1371/journal.pone.0117721

**Published:** 2015-03-10

**Authors:** Benjamin H. K. Yip, Roger Y. Chung, Vincent C. H. Chung, Jean Kim, Iris W. T. Chan, Martin C. S. Wong, Samuel Y. S. Wong, Sian M. Griffiths

**Affiliations:** Jockey Club School of Public Health and Primary Care, The Chinese University of Hong Kong, Hong Kong, China, Address: 4/F, School of Public Health Building, Prince of Wales Hospital, Shatin, Hong Kong, China; California Pacific Medicial Center Research Institute, UNITED STATES

## Abstract

**Objective:**

To examine the diagnostic performance of shorter versions of Alcohol Use Disorder Identification Test (AUDIT), including Alcohol Consumption (AUDIT-C), in identifying risky drinkers in primary care settings using conventional performance measures, supplemented by decision curve analysis and reclassification table.

**Study design and Setting:**

A cross-sectional study of adult males in general outpatient clinics in Hong Kong. The study included only patients who reported at least sometimes drinking alcoholic beverages. Timeline follow back alcohol consumption assessment method was used as the reference standard. A Chinese translated and validated 10-item AUDIT (Ch-AUDIT) was used as a screening tool of risky drinking.

**Results:**

Of the participants, 21.7% were classified as risky drinkers. AUDIT-C has the best overall performance among the shorter versions of Ch-AUDIT. The AUC of AUDIT-C was comparable to Ch-AUDIT (0.898 vs 0.901, p-value = 0.959). Decision curve analysis revealed that when the threshold probability ranged from 15–30%, the AUDIT-C had a higher net-benefit than all other screens. AUDIT-C improved the reclassification of risky drinking when compared to Ch-AUDIT (net reclassification improvement = 0.167). The optimal cut-off of AUDIT-C was at ≥5.

**Conclusion:**

Given the rising levels of alcohol consumption in the Chinese regions, this Chinese translated 3-item instrument provides convenient and time-efficient risky drinking screening and may become an increasingly useful tool.

## Introduction

The World Health Organization (WHO) indicated that alcohol consumption causes approximately 3.3 million net deaths globally in 2012, representing 5.9% of all global mortality [1]. Furthermore, alcohol-related harms accounted for 5.1% of the global burden of diseases and injuries, of which differed by sex: 7.4% for males and 2.3% for females [1]. The harmful use of alcohol is a causal factor in more than 200 diseases and injury conditions, such as alcohol dependence, liver cirrhosis and cancers [1]. Much of the harms of alcohol result from excessive consumption; however, these patterns of heavy consumption may not reach the threshold of alcohol abuse or dependence, as defined by the DSM-IV. A more common phenomenon, “at-risk drinking” or “risky drinking” is conceptualized as alcohol consumption levels that may put the drinker at risk for adverse health or social consequences [2]. According to the US National Institute of Alcohol Abuse and Alcoholism (NIAAA), risky drinking among non-elderly males is defined as consumption of ≥ 5 standard drinks (U.S. standard drink = 14g of ethanol) per occasion or averaging ≥ 15 standard drinks per week [2]. In the UK, the prevalence of risky drinking in both sexes who aged 15 years old or above was reported at 28.0% (males: 35.5%; females: 20.9%) [1]. In China, the annual drinking levels per person have been increasing rapidly in recent decades from 0.4 litres in 1952 to 2.5 litres in 1978, and to 4.9 litres in 2009 with concomitant rise in heavy drinking [3, 4, 5]. A recent study reported a 13% increase in cardiovascular deaths in a large Chinese population, which could be attributed to alcohol tax reduction [6]. These trends necessitate the development of valid screening tools for risky drinking in the general population in order to mitigate the harms of increasing alcohol consumption. Since it has been noted in the international literature that the prevalence of risky drinking is generally higher among patients attending primary care services as compared to the general population [7, 8], early detection and engagement of primary care in reducing risky drinking can be a cost-effective method of alcohol-related harms reduction [9]. The Alcohol Use Disorders Identification Test (AUDIT) recommended by the WHO is the most widely used alcohol misuse screening tool in the primary care clinical settings [10]. The AUDIT questionnaire consists of 10 items administered during a face-to-face interview. A meta-analysis revealed that AUDIT, with threshold scores varied between 4 and 8 from five studies, has good sensitivity (0.81, 95%CI: 0.75–0.86), specificity (0.88, 95%CI: 0.79–0.93), positive likelihood ratio: (6.62, 95%CI: 4.11–10.69) and negative likelihood ratio (0.21, 95%CI: 0.17–0.27) when compared to the reference standard. The reference standard was risky drinking; defined by quantity or frequency of consumption and heavy episodic drinking [11]. The WHO recommends that patients with a score of 8 or more points (out of a maximum 40 points) be given a brief intervention [12]. Subsequent diagnostic studies, however, employing reference standard for measuring risky drinking, have reported variable optimal cut-offs [13, 14].

The limited time for consultations in primary care is a major constraint for more widespread use of risky drinking screening. Primary care physicians were more likely to use shorter screening tool for alcohol screening [15]. In the search for simplified risky drinking screening tool, AUDIT has evolved over time from a 10-item screening questionnaire to several abbreviated versions. Common shorter versions include: a 3-item AUDIT Consumption (AUDIT-C) scale [16], a 2-item AUDIT Quantity-Frequency (AUDIT-QF) [17], a 1-item AUDIT-3 [18], a 5–item AUDIT Primary Care (AUDIT-PC) scale [19] and a 4–item Fast Alcohol Screening Test (FAST) [20].

The performance of abbreviated AUDIT as compared to its full version is traditionally determined on the basis of metrics such as sensitivity, specificity or area under the Curve (AUC). Despite the popularity of using these methods in diagnostic testing, there are increasing critics about their risk classification ability and their lack of clinical relevant [21–23]. Most importantly, comparing AUCs lacks an apparent clinical interpretation (e.g., does 5% increase in AUC justify clinical use of the new prediction model?). For AUDIT, the lack of reclassification performance measures hinders the choice between full and shorter versions, as their ability in correctly identifying risky drinking patients remains uncertain. For these reasons, other performance measures are encouraged [22, 24–25].

Reclassification has recently become popular approach for comparing improvement among risk prediction models for diagnosing common diseases [26–28]. A model is considered better when individuals who have subsequently developed the disease and those who have not developed the disease are reclassified to a higher risk category and to a lower risk category, respectively. The two recommended measurements of reclassification for quantifying improvement in risk prediction models are net reclassification improvement (NRI) and integrated discrimination improvement (IDI) which provide supplemental information over AUC [24, 29]. The decision-curve analysis quantifies clinical usefulness by displaying the net benefit (a weighted measure of true positive versus false positives) of using a prediction model across a range of diagnostic threshold [30]. For a binary test, markers risk levels are converted to a probability using logistic regression [23]. The diagnostic threshold chosen for decision making can be determined by literature review, expert consensus or clinical experience that takes into account harm-to-benefit ratio for a specific group of patients.

In China and Hong Kong, there has been interest in adopting greater screening and monitoring of drinking in clinical practice due to the rising rates of alcohol use [3, 4]. In this study, both conventional and recently developed performance measures were applied to compare the full 10-item Chinese language AUDIT with five different abbreviated versions of Chinese language AUDIT in detecting risky drinking in primary care settings. Our premise that if longer versions do not significantly improve the patients’ triage for receiving brief intervention or specialist care, then short versions could be used in Chinese speaking populations due to its higher implementability in the busy primary care settings. We tested *a priori* hypothesis that the shorter versions of Chinese language AUDIT had similar performance measures when compared with the full AUDIT.

## Materials and Methods

### AUDIT translation and validation

Two bilingual investigators preliminarily translated the original English instrument to the Cantonese version which was then examined for content validity by 15 native Cantonese speakers. The understanding and appropriateness of the wordings were discussed among the panel members. A modified Cantonese version was back-translated to English by another independent bilingual translator and this version was compared with the original English language AUDIT for equivalence. Based on the identified discrepancies, this process was repeated once more to produce a 10-item Chinese language that was subsequently pilot-tested on 28 male subjects who had reported ever consuming alcohol. Patients were then individually asked about the meaning of each item using a cognitive debriefing approach through face-to-face interviews. The investigators analyzed the pilot findings and a final 10-item Cantonese Chinese version was produced (Ch-AUDIT). Principal component analysis was used to investigate the psychometric properties of this final 10-item Ch-AUDIT. Similar to previous studies of AUDIT’s factor structure, the scree test indicated 3 factors could be extracted using criteria of Eigenvalue >1.0 and the total variance explained by these three components was 81%. The Ch-AUDIT also indicated good internal reliability (Cronbach’s alpha = 0.74, 95% CI:0.65–0.80).

### Participants and data collection

Data were collected by administering face-to-face interviews with a consecutive sample of 475 male patients attending public general outpatient clinics in Hong Kong. The general outpatient clinics are publicly funded primary care services [31], therefore patients are recruited from primary care setting. Only male were included in this study as female has a lower prevalence of alcohol problems than male do [3]. The clinics were situated in a territory which has similar socio-demographic characteristics with the entire Hong Kong population. Patients who met the following criteria were eligible for the study: (i) age≥ 18, (ii) responded affirmatively to the pre-screening question recommended in the NIAAA Clinician’s Guide (“Do you sometimes drink alcoholic beverages?”), and (iii) able to provide written informed consent. Patients with limited Cantonese language ability, cognitive impairment or acute illness were excluded. We followed the QUADAS-2 recommendation of recruiting consecutive patients without setting any additional exclusion criteria based on age or diagnosis, as this will contribute to exaggerated diagnostic accuracy [32]. The Ch-AUDIT was administered in a face-to-face interview and data were recorded anonymously without any identifying information. To minimize social desirability bias, the interviewer assured the patients that the information collected will not be conveyed to their attending doctors and allied health professions in the clinic. Ethical approval was obtained from the clinical research and ethics committee of the sponsoring university. All ethical safeguards in accordance with the Declaration of Helsinki have been met.

### Reference standard

After the completion of Ch-AUDIT interview, the participant’s alcohol consumption was assessed by the calendar-based timeline follow back (TLFB) approach. Using the TLFB approach, subjects will estimate the quantity of standard drink alcohol consumed on each of the 30 days preceding the interview. This approach serves as a validated reference standard as subjects estimated the amount of alcohol consumed on each of the 30 days preceding the interview [33]. Average weekly alcohol intake was calculated by multiplying the average number of standard drinks consumed per day during the 30 days by 7. Subjects will be considered to have risky drinking behavior if their consumption reached the NIAAA recommended thresholds [2].

### Statistical analysis

To evaluate clinical usefulness of the short versions of Ch-AUDIT, including AUDIT-C, AUDIT-QF, AUDIT-3, AUDIT-PC and FAST, we compared their performance with the Ch-AUDIT. The AUDIT-C scale (comprised of items 1, 2 and 3 of the original AUDIT) [16], AUDIT-QF (items 1 and 2) [17], AUDIT-3 (item 3) [18], AUDIT-PC scale (items 1, 2, 4, 5 and 10) [19] and FAST (items 3, 5, 8 and 10) [20] were compared. For each version of Ch-AUDIT, the items were treated as the predictors in a logistic regression with risky drinking as the binary outcome. Akaike information criteria, Brier score and Nagelkerke *R* ^2^ were used to assess the overall model performance. We used Hosmer-Lemeshow test to assess calibration of the prediction models by dividing subjects into deciles of risk and compared the predicted risk with the actual proportion of outcomes in each decile [34]. AUC was used to indicate the discriminative ability of the prediction models. A non-parametric approach was used to test the difference in AUC [35]. A shorter version of Ch-AUDIT was considered promising if the difference in AUC was not statistically significant when compared with Ch-AUDIT. Statistical significance was set at α = 0.05.

We conducted decision curve analysis to calculate the net benefit of the different versions of Ch-AUDIT [30]. Net benefit (NB) is calculated across the full range of threshold probabilities (0%-100%). The threshold probability (*p*_t_) is the cut-off probability that determines a positive or negative result for clinical decision. Net benefit is a weighted measure for detecting disease (true positives, TP) versus over-diagnosing non-disease (false positives, FP), defined as Net benefit = (TP-wFP)/*N*, where *N* is the total number of subjects, and *w* is the relative weight, calculated by*p*_t_/(1-*p*_t_) [29, 30]. A high net benefit indicates a good prediction model. An optimal threshold of *p*_t_ should be independent of a data set and concerns only the consequences of true and false positives [25]. For instance, if missing a risky drinking case (false negative) is considered to be four times worse than a false positive then this is an odd of 4 to 1, or a threshold probability of 20%. Given one or a range of targeted threshold probabilities could give the corresponding scores of that particular version of Ch-AUDIT, which facilitates the search of an optimal cut-off score. In the context of this study, unnecessary brief intervention or referral for a non-risky drinker should be avoided, but is less problematic than withholding these interventions among risky drinker. The threshold of 25% reflects a 1:3 relative weights of these errors, which can be considered for conditions that do not pose immediate serious health consequence to patients.

Given a threshold probability, different versions of AUDIT were further compared for their reclassification ability by using NRI. Following the definition of NRI, Ch-AUDIT serves as the “old” model and other shorter versions of Ch-AUDIT as the new/test model. When the new model reclassified a subject with the outcome (risky drinker) into higher risk group implies improved classification. The interpretation is opposite for subjects without the outcome (e.g., non-risky drinker). NRI is the sum of differences in proportions of correct reclassification minus incorrect reclassification. A positive NRI reflects the likelihood that risky drinker subjects were more likely to move up a category (e.g., as high-risk) than down (e.g., as low-risk) when compared to non-risky drinker subjects. IDI integrates net reclassification over all possible threshold probabilities and is equivalent to the difference in discrimination slopes between the new and old model [29]. The relative change of IDI will be reported, where a positive value indicates the new model improves the discrimination.

Finally, we also examined the optimal cut-off point of each promising version of AUDIT by using the conventional statistics, including sensitivity, specificity and balanced accuracy. All analyses were performed using R version 2.15.2 (R Foundation for Statistical Computing; www.r-project.org) with the pROC and PredictABEL packages.

## Results

The socio-demographic characteristics of the respondents were summarized in Table 1. Among the 475 study participants, 46.1% were between 50–64 years of age and 27.6% were equal to or over 65 years of age. The majority (81.7%) reported educational attainment of secondary school or below. Using the TLFB approach, the estimated proportion of risky drinking in this sample was 21.7% (n = 103).

**Table 1:** Characteristics of Subjects Study Sample (N = 475).

Characteristic	N	%
Age group		
15–29	31	6.5
30–49	92	19.4
50–64	219	46.1
≥ 65	131	27.6
Missing	2	0.4
Education		
Primary or no education	125	26.3
Secondary education	263	55.4
Tertiary education	81	17.1
Others	6	1.3
Marital status		
Married	407	85.7
Single	51	10.7
Others	17	3.6
Occupation		
Employed	258	54.3
Unemployed	22	4.6
Retired	172	36.2
Other	23	4.8
Alcohol Measures		
Average Weekly Alcohol consumption > 14	45	9.5
Daily limits > 4	84	17.7
Risky drinker	103	21.7
Non-Risky drinker	243	51.2

### Overall performance, calibration ability and discrimination ability

Ch-AUDIT had the best overall performance (highest Nagelkerke’s *R* ^2^ and lowest Brier score), good calibration as assessed by the Hosmer-Lemeshow test (p-value = 0.181) and high discrimination ability (AUC = 0.901) (Table 2). Nevertheless, while the Ch-AUDIT was comprised of more items, this did not result in a significantly improved discriminative ability as compared to some abbreviated versions. The AUC of 10-item Ch-AUDIT only improved by 0.016 when compared to that of the 3-item AUDIT-C instrument. The FAST and AUDIT-3 instruments showed AUCs that were significantly different from the Ch-AUDIT and of the remaining three instruments, the 5-item AUDIT-PC did not demonstrate significantly improved performance over the shorter 3-item AUDIT-C or the 2-item AUDIT-QF.

**Table 2:** Overall performance of abbreviated AUDIT compared to Ch–AUDIT.

	Number of items	*R* ^2^	Brier Score	Area under the Curve (AUC)	p-value*
Ch-AUDIT	10	0.577	0.084	0.901	—
AUDIT-PC	5	0.533	0.090	0.884	0.185
FAST	4	0.434	0.107	0.842	0.007
**AUDIT-C**	**3**	**0.558**	**0.088**	**0.898**	**0.959**
AUDIT-QF	2	0.532	0.090	0.881	0.110
AUDIT-3	1	0.418	0.109	0.823	<0.001

*DeLong approach was used to compare the ROC curve of the Full 10-item Chinese language AUDIT with other abridged versions of AUDIT shown.

### Decision curve analysis

**Fig 1:**
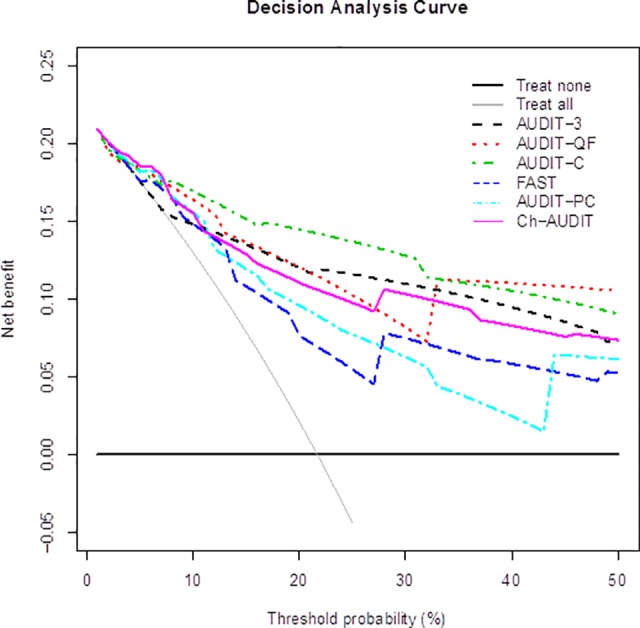
Decision curves showing net benefit in comparisons between various versions of Ch-AUDIT.

The decision curves in Fig. 1 demonstrate that all models were never worse than the most sensitive strategy of treating all patients (gray solid line) and also better than the most specific strategy of treating none (black solid horizontal line), at least up to a *p*_t_ of 50%. FAST and AUDIT-PC had lower Net benefit than Ch-AUDIT when *p*_t_ is between 5% and 50%. All consumption-related versions of Ch-AUDIT (AUDIT-3, AUDIT-QF, and AUDIT-C) had in general higher net benefit than Ch-AUDIT. AUDIT-C had consistently highest net benefit, particular in the range between 15%-30%. If *p*_t_ is larger than 32% AUDIT-QF is preferable. The largest discrepancy in net benefit between AUDIT-C and Ch-AUDIT was at the threshold probability of approximately 25%, which implied a relative weight of 1:3 for false-positive decision against true-positive decision.

### Optimal cut-off scores

At this threshold, the possible cut-off of AUDIT-C should be a score of ≥4 or ≥5; and for Ch-AUDIT the cut-off should be a score of ≥7 or ≥8, revealed by the regression estimated risk. The negative predictive values (NPV) of Ch-AUDIT score of 7 or 8 were good (>0.90), but the positive predictive values (PPV) were all less than 0.5 and the balance accuracies were all more than 0.70 (Table 3). By contrast, AUDIT-C with a cut-off score of 5 had the best-balanced accuracy (0.83), high NPV (0.93) and a moderate level of PPV (0.64).

**Table 3:** Diagnostic accuracy of Ch-AUDIT and AUDIT-C across different cut off scores for screening of risky drinking behavior among male patients in primary care setting.

Version	Cut-off score	Sensitivity	Specificity	PPV	NPV	BalancedAccuracy
Ch-AUDIT	≥ 5	0.903	0.535	0.350	0.952	0.719
	≥ 6	0.825	0.637	0.386	0.929	0.731
	≥ 7	0.748	0.737	0.440	0.913	0.742
	≥ 8	0.680	0.809	0.496	0.901	0.744
AUDIT-C	≥ 4	0.922	0.648	0.420	0.968	0.785
	**≥ 5**	**0.777**	**0.879**	**0.640**	**0.934**	**0.828**
	≥ 6	0.621	0.944	0.753	0.900	0.782

Key: PPV = Positive predictive values, NPV = Negative predictive values.

### Reclassification

To better integrate sensitivity and specificity, and to compare the classification ability between the two better performing versions of Ch-AUDIT, reclassification table for cases (risky drinkers) and controls (non-risky drinkers) were created respectively (Table 4). In total, 141 males were classified as high risk using Ch-AUDIT, and 125 males were classified as high risk using AUDIT-C. Among non-risky drinkers, AUDIT-C incorrectly reclassified 6% (18/301) as risky drinker, but correctly reclassified 62% (44/71 respondents) as non-risky drinker compared to Ch-AUDIT. Among risky drinking patients, AUDIT-C correctly reclassified 39% (13/33) respondents as a risky drinker, but only incorrectly reclassified 4% (3/70 subjects) as a non-risky drinker. In total, AUDIT-C reclassified 16.4% (78/475) of all respondents. The NRI value was 0.167 (95% CI: 0.0826–0.2513), indicating that risky drinker were approximately 16.7% more likely to be classified as high-risk compared to non-risky drinker by using AUDIT-C compared to Ch-AUDIT. IDI was 0.106 (95% CI: 0.063–0.149), which implies that AUDIT-C on average improved the discrimination by 10.6% compared to Ch-AUDIT.

**Table 4:** Reclassification performances of Ch-AUDIT and AUDIT-C for identifying risky drinking male patients in primary care setting.

Non-Risky drinkers					
			AUDIT-C		
			Low risk (<25%)	High risk (≥25%)	Reclassified (%)
	Ch-AUDIT	Low risk (<25%)	283	18	6
		High risk (≥25%)	44	27	62
Risky drinkers					
			AUDIT-C		
			Low risk (<25%)	High risk (≥25%)	Reclassified (%)
	Ch-AUDIT	Low risk (<25%)	20	13	39
		High risk (≥25%)	3	67	4
Total sample					
			AUDIT-C		
			Low risk (<25%)	High risk (≥25%)	Reclassified (%)
	Ch-AUDIT	Low risk (<25%)	303	31	9
		High risk (≥25%)	47	94	33

## Discussion

In this study, we used both conventional and novel performance measures to compare various versions of Ch-AUDIT in their ability to identify risky drinking in primary care setting. AUDIT-C was the most promising short version of the original AUDIT in discrimination (AUC = 0.898) and overall performance (Brier score = 0.0882). The decision curves analysis reveals that AUDIT-C has the largest net benefit value when the threshold probability is set within the reasonable range from 10% to 30%. This study suggests that AUDIT-C screening cut-off should be set at ≥5 to maximize sensitivity, specificity, PPV and NPV. Moreover, as clearly displayed by the reclassification table, AUDIT-C has 16.7% better ability on average in identifying risky drinking cases than Ch-AUDIT.

Our findings are consistent with a meta-analysis of five cross-sectional studies comparing AUDIT-C against risky drinking in primary care setting (n = 8679) [11]. The meta-analysis reported a sensitivity of 0.97 (95%CI: 0.90–0.99), specificity of 0.68 (95%CI: 0.56–0.77), positive likelihood ratio: 2.99 (95%CI: 2.22–4.03), and negative likelihood ratio: 0.04 (95%CI: 0.01–0.14). There were also no statistically differences between overall accuracy of the AUDIT and the AUDIT-C. Two studies conducted in community settings were published subsequent to this meta-analysis, with better performance in terms of specificity [33, 36], but tailored cut-off points according to specific group (e.g., age, gender) were recommended. Another systematic review revealed the general optimal cut-off point was ≥ 6 for male and ≥ 5 for female [13 ]. Whereas, in this study the optimal cut-off point of AUDIT-C is ≥ 5, which might be explained by lower alcohol consumption in Hong Kong [3]. The lower threshold might also be explained by the exclusion of non-drinkers in the present study (which typically improves specificity). Recently, a South Korean study suggested using AUDIT-5 (5-items) as the universal brief screening test for risky drinking, alcohol use disorders and alcohol dependence [37]. However, for population with low prevalence of problematic drinking, AUDIT-5 may not be the optimal for use in a primary care setting. For other settings with higher prevalence of alcohol abuse or dependence, as comparable to South Korea, AUDIT-5 might be a more optimal choice. It should be highlighted that variations in psychometric properties and optimal cut off scores of AUDIT across studies could also be attributed to drinking patterns of those who received screening and gender. For policy purposes of deciding an optimal cut-off point of AUDIT-C, we must consider the prevalence of risky drinking in the setting; and the cost of false positive relative to the benefit of true positive screening, including the actual cost of using an abbreviated 3-item instrument versus the full 10-item version.

A cost effectiveness modeling study on UK health system highlighted that screening all primary care attendees with full AUDIT rather than AUDIT-C offers a slightly improved sensitivity, but the incremental cost-effectiveness ratio of £62,000 per QALY gained suggests that using the full questionnaire is not cost-effective [38]. Another consideration is who should conduct the screening. A systematic review of 17 USA-based studies reported that the cost per screen ranges from US$ 0.51 to $601.5, with a median cost of US$ 4 (2009 US dollars). The cost tends to be lower when doctors are not administering the questionnaire [39]. In UK, it can be assumed that the cost of screening by a nurse is £0.55 per minute, while for GP the cost would raise up to £2.72 per minute [40]. In view of such cost, replacing AUDIT with AUDIT-C may reduce screening time from 10 minutes to 1–2 minutes, which will remove a major barrier to the implementation of risky drinking screening in busy clinic settings [39].

Compared to previous studies, a major strength of the present study was the utilization of more up-to-date performance measures to conduct head-to-head comparisons between the different AUDIT versions. However, this study has several limitations. First, we could only study one single aspect of problematic drinking, which is risky drinking behavior as defined by TLFB, and we could not conduct analysis on other alcohol problems, such as alcohol abuse or alcohol dependence. Nevertheless, the prevalence of alcohol abuse or dependence has traditionally been low in Hong Kong, as well as in China (4.9% for alcohol use disorders and 2.3% for alcohol dependence in both sexes in 2010) [1, 3, 41]. Second, the AUDIT was administered with data recorded anonymously, which limits the generalizability to screening results obtained in routine clinical care. Third, the instruments we employed limit our findings of this study. TLFB is designed to assess alcohol consumption levels; thus it may not be a surprise that the short versions with primarily consumption items (i.e., AUDIT-C and AUDIT-QF) had the best performance among other versions of AUDIT. Again, due to the low prevalence of alcohol abuse and dependence, other reference standards, such as the DSM criteria, are less optimal for detecting risky drinking behavior. Third, this study only focused on men, but not on women. But again, the prevalence of alcohol problems has been low in Hong Kong, and even lower so for women [3, 41].

## Conclusions

This is the first study that compares the diagnostic performance between various shorter versions of AUDIT using decision curve analysis and reclassification table. The study sample included only male primary care patients who reported at least sometimes drinking alcoholic beverages. In conclusion, our findings confirm the validity of several short versions of the AUDIT, specifically AUDIT-C. This 3-item instrument provides a convenient and time-efficient screening of risky drinking in primary health care setting. We recommend a cut-off (≥5) score of AUDIT-C for populations with low prevalence of alcohol abuse and dependent, such as Hong Kong, to identify risky drinkers. Given the rising levels of alcohol consumption in the Chinese regions, the Chinese translated and validated abbreviated versions may become increasingly useful tools in primary care practice for reducing the harms of alcohol use.
